# Investigational and Numerical Examination on Bending Response of Reinforced Rubberized Concrete Beams Including Plastic Waste

**DOI:** 10.3390/ma16165538

**Published:** 2023-08-09

**Authors:** Fuat Korkut, Memduh Karalar

**Affiliations:** 1 Department of Civil Engineering, Van Yuzuncu Yıl University, 65080 Van, Turkey; 2 Department of Civil Engineering, Zonguldak Bulent Ecevit University, 67100 Zonguldak, Turkey; memduhkaralar@beun.edu.tr

**Keywords:** bending behavior, finite element analysis, cracking attitude, reinforced concrete beam, plastic waste

## Abstract

In this investigational study, the fracture and bending performance of reinforced concrete beams (R-C-Bs) with varying proportions of plastic waste (PW), considered as fine aggregate (FA), were assessed via experimental and numerical examination. To achieve this aim, altered concrete series were designed, with the aggregate sizes changed within the range of 0 to 25 mm. To enhance the concrete, PW was selected to be used in combination with aggregate material measuring 0 to 5 mm in particle size, as an alternative FA, with proportions of 0%, 5%, 15%, 30%, and 45%. Experiments were performed to examine the performance of the R-C-Bs. It was found that a 30% PW proportion offered the optimum results in terms of displacement capability. Furthermore, ANSYS v.19 software was chosen to form 3D finite element models (F-E-Ms) of R-C-Bs to be compared with the experimental data. The experimental and 3D F-E-M investigations offered remarkably close-fitting bending and rupture performances. Then, a structure was modeled using SAP2000, and the strength of the R-C-Bs was then used in an RC structural model. The results show that the forces on the construction caused reductions while also increasing the PW proportion. Moreover, it was realized that the F-E-M simulations and experiments produced tiny cracks with highly matched formations.

## 1. Introduction

The amount of synthetic plastic consumed in the world is increasing steadily every year. Increasing synthetic plastic consumption might have occurred due to the practical features of synthetic plastic, specifically, factory manufacture, the weightlessness of plastic products, and the low manufacture price [1,2]. Plastic is widely used in bottles, food packaging, industrial goods, communication supplies, and enclosures, among other usages. Though numerous techniques have been chosen for the removal of synthetic trash, most treatments are insufficient, because of excessive synthetic waste generation. For this reason, another possibility is to reuse synthetic trash and customize it as fiber reinforcement for concrete. Nowadays, synthetic fibers are popular for strengthening lightweight precast concrete components such as double walls, pipes, and sleepers. In earlier investigations, it was noted that these uses might successfully regulate cracks [3,4,5,6] and prevent dry shrinkage cracks in concrete [7]. Ramadevi and Manju [8] inspected the probability of using waste bottles in the restricted alteration of aggregate in Portland cement. For this purpose, in their investigation, concrete with 1%, 2%, 4%, and 6% bottle fibers as FA was manufactured and compared with a control combination with no alteration. At the end of this investigation, Ramadevi and Manju [8] found that there was an increase in compression and tensile strength; therefore, bottle fiber alterations might be implemented to enhance concrete. Another investigation was performed by Baldenebro-Lopez et al. [9]. In this investigation, Baldenebro-Lopez et al. [9] provided results from a theoretical–experimental program of partly pre-stressed R-C-Bs, prepared with continuously reused PET strip-reinforced concrete. In this investigation, the load–deflection, ductility, and energy absorption ability of the R-C-Bs were detected, and these factors predicted the flexural performance of the R-C-Bs. At the end of the tests, the results obtained were associated with computer examination and investigational data to verify the validity of the proposed technique, demonstrating that the theory also calculates the post-cracking creep deformation effectively. Another investigation was performed by Nibudey et al. [10]. In this investigation, the direct shear performance of R-C-Bs including PET waste fibers was noted. For this purpose, PET fibers were used in plain concrete, in quantities up to 3% at an increase of 0.5%, with characteristic proportions of 35% and 50%. At the end of the investigation, it was found that a shear improvement of 27.25% was associated with the addition of 1% of fiber, with a characteristic proportion of 50%. Jahami et al. [11] inspected the load deformation performance of lightweight R-C-Bs using the calculations recommended by Branson [12,13] and Bischoff [14]. At the end of this investigation, Jahami et al. [11] offered a newly established calculation and PW was proposed. Khatiba et al. [15] examined the structural ability of R-C-Bs containing PW. For this purpose, the PW was obtained from the cap of a plastic bottle. The coarse aggregate was altered with 0, 10%, 15%, and 20% (by volume) PW. At the end of the investigation, it was found that the first rupture performed on the control beam was at a load of 56 kN, and it regularly increased with rising PW content, reaching 66 kN at 20% PW alteration. This was a good indication of the PW content in concrete postponing the presence of a rupture. Furthermore, as for the strain values, the tensile strain in the control beam was greater for the same load than that in the beams with PW content. Diana et al. [16] selected plastic bottle waste as a substitute for FA, to have sand-like gradation. For this purpose, the variants of graded plastic bottle waste were set at 0%, 5%, 10%, and 12%. It was found that the higher the PW content, the poorer the concrete strength (CS). The maximum CS occurred at the variants of 0% and 5%, specifically, with 19.192 MPa for the combination of 0% and 16.414 MPa for the combination of 5%. In general, concrete that includes more plastic has a poorer permanency performance. Ismail and AL-Hashmi [17] performed 86 experiments and 254 tests to define the productivity of reprocessing PW in the manufacture of concrete. These examinations contained slump, fresh density, dry density, CS, flexural strength, and toughness indices. At the end of this investigation, it was found that reprocessing PW as a sand-substitution aggregate in concrete provides a good approach to decreasing the price of constituents and solves some of the solid-waste difficulties posed by plastics. Ibrahim et al. [18] performed investigational examinations to observe the performance of concrete’s mechanical properties, using R-C-Bs that contained fine PW aggregate as a partial substitute for natural sand aggregate. For this purpose, six concrete combinations were designed with different fine PW aggregate amounts, which were set as 0%, 25%, 50%, 75%, and 100%, along with the addition of polyvinyl alcohol with the 75% fine PW aggregate amount. At the end of the investigation, it was found that using the fine PW aggregate in the concrete impacted the concrete performance by reducing workability and flexural, shear, tensile, and overall concrete strength. Gesoglu et al. [19] investigated PW powder as a cement alteration material and the mechanical and rupture characteristics of self-compacting concretes containing different quantities of PW powder. For this purpose, partial quantities of cement were swapped with PW powder at 5%, 10%, 15%, 20%, and 25% by weight. Then, the failure features of the concrete were noted using the three-point deformation test on the R-C-Bs. The conclusions showed that the mechanical properties of PW-powder-modified self-compacting concrete reduced as the concrete became less brittle. Mohammed and Rahim [20] defined the material properties of high-strength concrete with changed waste fiber in terms of fiber volume and length. The structural performance of R-C-Bs prepared using this newly developed concrete was also examined by Mohammed and Rahim [20]. It was found that the cracking performance of R-C-Bs increased as a consequence of using waste fiber. On the other hand, it was observed that there was no rise in ultimate charge ability.

There are several examinations on concrete performance using waste supplies [21,22,23,24] and other materials, such as composite [25,26,27,28,29,30,31], in the literature. Furthermore, it is known that the creep and shrinkage behavior of R-C-Bs is significant for the durability of constructions [32,33,34]. Investigators have recognized changes in the properties of concrete, but limited studies have discussed the trade-offs of using a fine natural aggregate prepared in a typical Portland cement (PC) combination. The gap in the literature is represented by the determination of an optimum PW ratio to replace FA in reinforcement concrete, and accordingly, there are almost no investigations on the weight of a reinforced concrete structure. This investigation supports several notions in the literature.

## 2. Purpose of Investigation

The influence of PW on the crack and flexural performance of R-C-Bs was observed in this investigation. For this purpose, five different R-C-Bs (300 × 400 × 2000 mm) were prepared in the lab, and five different PW proportions were used in these R-C-Bs. These PW proportions were chosen to replace 0–5 mm grain size aggregates in the following amounts: 0%, 5%, 15%, 30%, and 45%. The influence of numerous PW proportions on the consistency of fresh concrete was first examined. To achieve this aim, five separate slump examinations were conducted for these PW proportions. At this point, to make standard cube examples, 15 different concrete examples (CEs) with altered PW proportions (three CEs for each PW proportion) were designed in molds with dimensions of 150 × 150 × 150 mm. These cube examples were kept in water for 28 days to cure. The R-C-Bs’ mechanical properties were obtained. These examples were subjected to a CS examination to define their CS. One of the key aims of this investigation was to observe how different PW proportions affect the rupture (flexural strength is used to assess rupture) and deformation performance of R-C-Bs. The calculation of deformation is very significant in R-C-Bs as it represents a very important serviceability check in the design of constructions. If deformation is excessive or extends further than the permissible borders, it might lead to cracks, destruction to finishes, and the misalignment of structure services/fittings. Thus, in the research lab, the R-C-Bs produced in this way were exposed to deformation and cracking examinations. Furthermore, in this investigation, 3D numerical examinations were conducted and confirmed as a result of comparing experimental data. For this purpose, ANSYS [35] was chosen to model the 3D F-E-Ms of the R-C-Bs. Deformation and rupturing were achieved in the 3D models of R-C-Bs via numerical analyses, and these cracks and curves were designed to be similar to investigational rupture data. This investigation then assessed the effect of the PW alteration proportion on the weight of constructions. The influence of these PW proportions on the shear and moment forces formed on the elements of a structure were modeled in detail using SAP2000 [36] software.

## 3. Investigational Examination Set-Up

### 3.1. Examination of Material Properties

**Figure 1:**
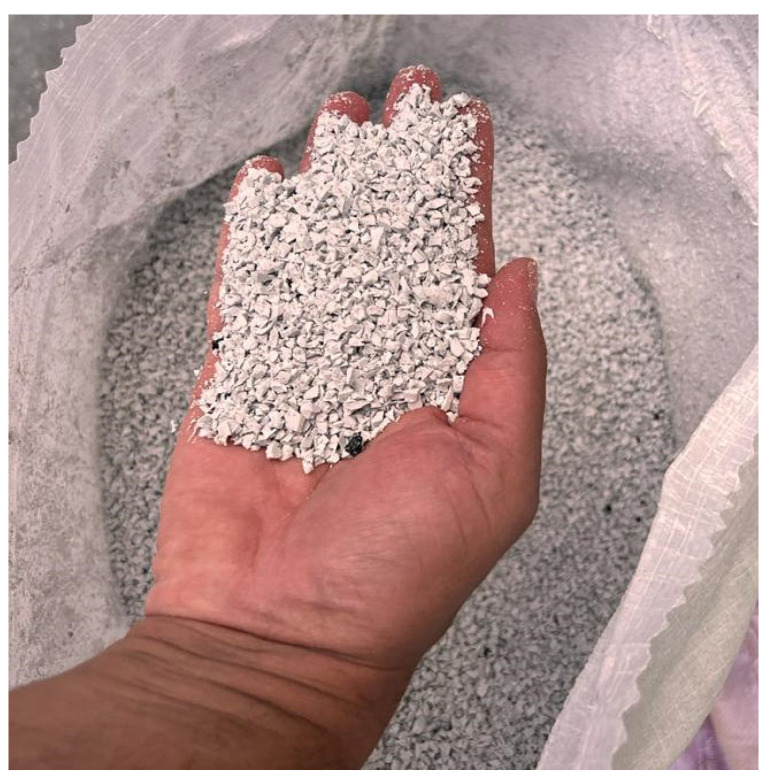
Photograph of the plastic waste.

Defining the existing and potential protection of constructions that use reinforced concrete by investigating rupture and deformation in the R-C-B is vital. PW in a variety of proportions was reused subject to the proportion of PW in individual R-C-Bs. A photograph of the plastic waste is shown in Figure 1. It was essential to define the concrete’s mechanical properties using lab examinations. The results of the experiments, presented in Table 1, feature the specific concrete combinations that were prepared in the lab. The investigational examination methods included the use of several proportions of plastic waste: 0%, 5%, 15%, 30%, and 45%. PW was chosen in the concrete preparations to replace aggregates with a 0–5 mm particle size. The mixing proportions are described in Table 2.

**Figure 2:**
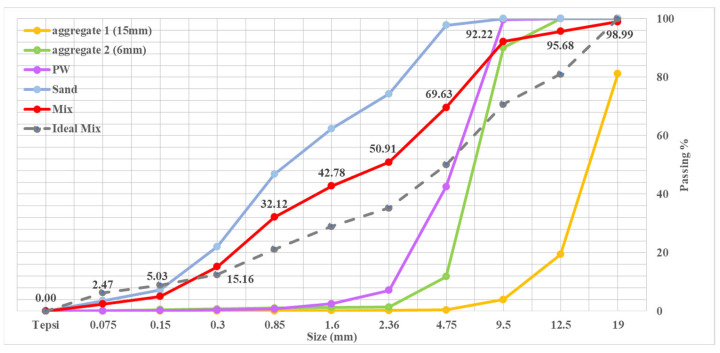
Subdivision dimension distribution of the aggregates.

The Table 2 demonstrates that the reference concrete contains no plastic waste, and an improvement in the proportion of PW from 0% to 45%. Pozzolanic cement that uses CEM IV/B (P) 32.5 N/R was chosen for the concrete examples. Table 3 shows the cement structure. Furthermore, local tap water in Ankara was used. The cement was replaced by PW with weight proportions of 5%, 15%, 30%, and 45%. A superplasticizer was used in the concrete combinations to both decrease the quantity of water required and improve the permanency of the concrete. The PW used as the fine material at the OSTIM came from Ankara, Turkey. While there were five examples of altered concrete, the reference concrete’s maximum unit weight was 492 kg, and the 100% PW proportion had a maximum unit weight of 648 kg. Next, slump examinations were conducted for each concrete combination. The subdivision dimension distribution of the aggregates is shown in Figure 2.

To define the CS of concrete including plastic waste, the concrete examples were poured into a mold with dimensions of 150 × 150 × 150 mm. A total of 15 different concrete examples (CEs) were produced via standard cube examples obtained from five concrete examples with different proportions of PW alteration. They were kept for 28 days at temperatures of 20–22 degrees Celsius. Then, the concrete examples were removed from the molds, and their CSs were noted. To analyze 3D cracking in R-C-Bs, one is required to employ CS and concrete grades. Pressure tests were conducted according to the TS EN 12390-3 standard. Based on this standard, a fixed load of 0.6 (N/mm^2^·s) was applied when carrying out compressive tests. The load could be applied to the example at a continuous speed until the maximum load was reached, without the influence of impact, so that the deviation from the chosen speed did not exceed ±10%. Furthermore, the CS outcomes for each concrete example are shown in Table 4.

### 3.2. Investigational Set-Up

**Figure 3:**
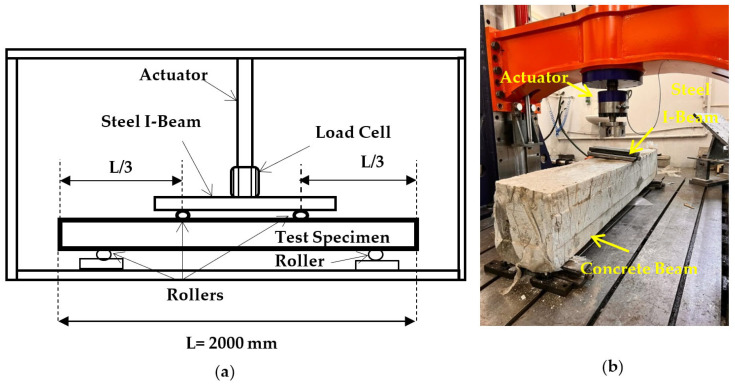
(**a**) Loading arrangement for static testing; (**b**) static testing device.

The investigational R-C-B set-up is defined in this section. To perform the R-C-B investigation, a total of two reinforcements were used for compression reinforcements at the top section of the R-C-B, and three reinforcements were chosen for tension reinforcements at the bottom of the R-C-B. Furthermore, 12 mm diameter reinforcements were also chosen as the compression and tension reinforcements. In addition, 8 mm diameter reinforcements were used for the stirrups. Figure 3 demonstrates the test set-up. Figure 3 shows that the R-C-B was held by one pin and one roller. The L-V-D-T used to record the deformation of the R-C-Bs was ±250 mm long, as shown in Figure 3. A load-cell, a device that can measure a ton’s worth of weight, was also used to test the strength of the R-C-Bs. Furthermore, an investigational flowchart is presented in Figure 3. The perpendicular load and displacements used were calculated using a data-logger and kept on a hard disk associated with the device through a PC [25].

## 4. Investigational Test Results and Discussion

In this section, the influence of the PW on the cracking and deformation performance of the R-C-Bs is noted. For this purpose, the R-C-Bs that were manufactured in the lab were tested to assess their cracking and deformation performance. The R-C-Bs were noted to be disrupted at multiple locations and their positions were closely examined, with each breaking pattern being unique. Deformations were observed in each R-C-B’s results, and these may be related to the different PW proportions used. The R-C-Bs were loaded separately, which was important for examining the influence of PW proportion on the R-C-Bs’ cracking and deformation performance. Table 1 shows that five separate R-C-Bs were originally created in this investigation.

### 4.1. Case 1: Cracking and Deformation Performance of R-C-B without Plastic Waste

**Figure 4:**
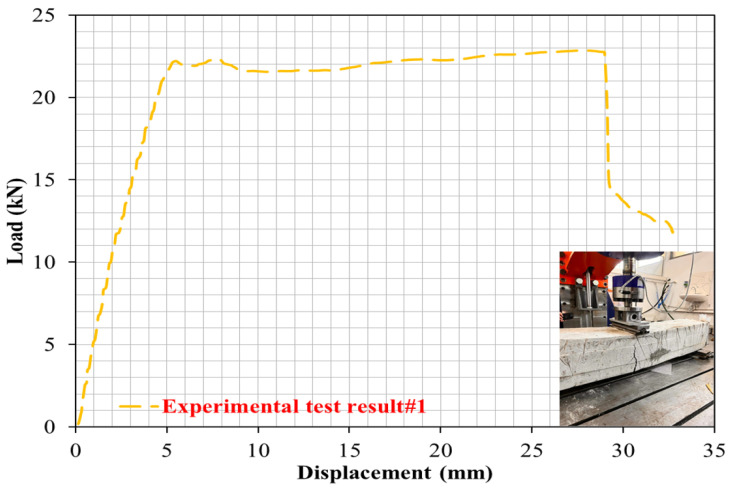
Load deviation performance of the reference R-C-B under perpendicular loads.

**Figure 5:**
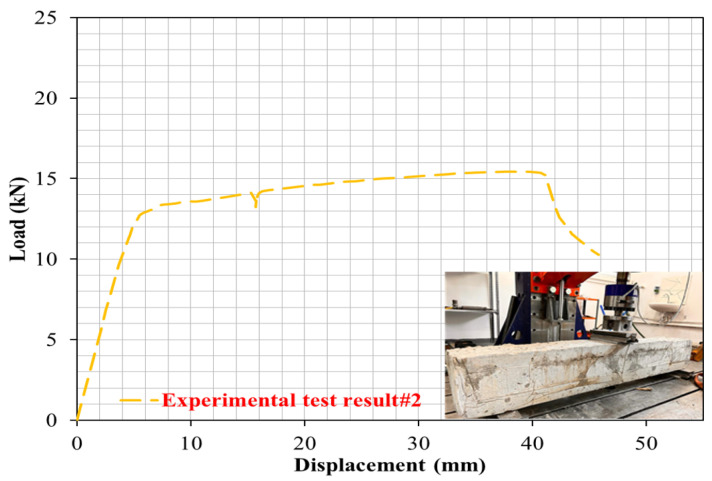
Load deviation performance of the R-C-B including 5% plastic waste.

The influence of the proportions of PW on the cracking and deformation performance of the R-C-Bs was considered. For this purpose, the proportion of PW in the R-C-B was set as 0%. As detailed by the empirical values, there was an important shear crack in the reference R-C-B subjected to the perpendicular load. According to Figure 4, it was detected that the largest shear crack started from the place in which the perpendicular load was applied to the R-C-B, and there was cracking behavior toward the bottom of the R-C-B. Load–displacement curves were obtained for the R-C-Bs, as shown in Figure 5. These were calculated using an L-V-D-T device, and the results are reported. At the end of the investigational test, a 3.96 cm crack occurred in the R-C-B. The results show the size of the rupture gap that can be expected for perpendicular loading. These observations indicate that a significant break can occur in R-C-Bs when a heavy load is present and dangerous deformation cracking occurs. Perpendicular cracks were common in the R-C-B, as this situation exhibited. More significantly, the reference R-C-B had a separate perpendicular rupture of 150 mm. The positions of the maximum flexural cracks were clearly visible: above the R-C-B where the load was applied, and then, below the R-C-B. In Figure 4, the reference R-C-B load–deflection curve is shown. According to the results in Figure 4, the maximum deflection was 33.9 mm, while the R-C-B was unable to hold any more weight. Therefore, the R-C-B can carry a load of 228.9 kN and display bending of 38.9 mm under a perpendicular load. The results presented normally contain a wealth of information about the load-carrying ability of reference R-C-Bs.

### 4.2. Case 2: Cracking and Deformation Performance of R-C-B Including 5% Plastic Waste

**Figure 6:**
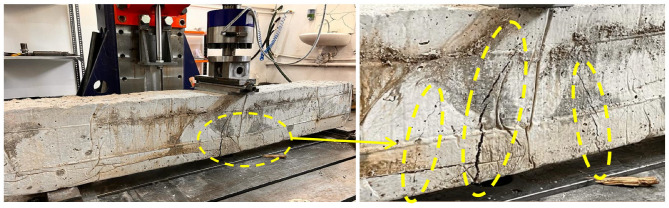
Cracking progress of the R-C-B including 5% plastic waste.

To observe the influence of the proportions of PW on the cracking and deformation performance of the R-C-Bs, the proportion of PW in the R-C-B was set as 5%. As can be seen in Figure 5, when the PW amount in the combination was enhanced from 0% to 5%, there was a 33% decrease in the load-carrying ability of the R-C-B, whereas the deformation rate increased by 32%. Figure 5 shows that important cracking occurred in R-C-Bs under strong loads, with noteworthy deformation cracking. The positions of these ruptures were where perpendicular cracks might take place in the R-C-B. The load-carrying ability of this R-C-B was found to be 15.5 kN. The corresponding deformation amount was 44 mm. The cracking patterns are shown in Figure 6. The reason for this condition was that cracking occurs more quickly as a result of the bearing ability of the R-C-Bs.

### 4.3. Case 3: Cracking and Deformation Performance of R-C-B Including 15% Plastic Waste

**Figure 7:**
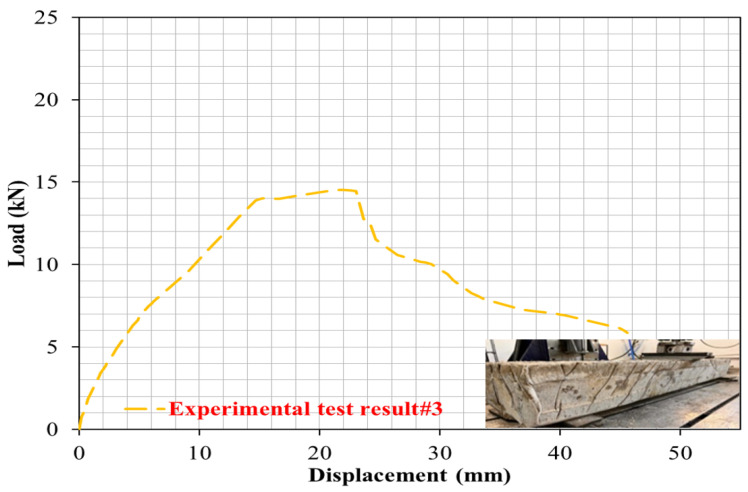
Load deviation performance of the R-C-B including 15% plastic waste.

**Figure 8:**
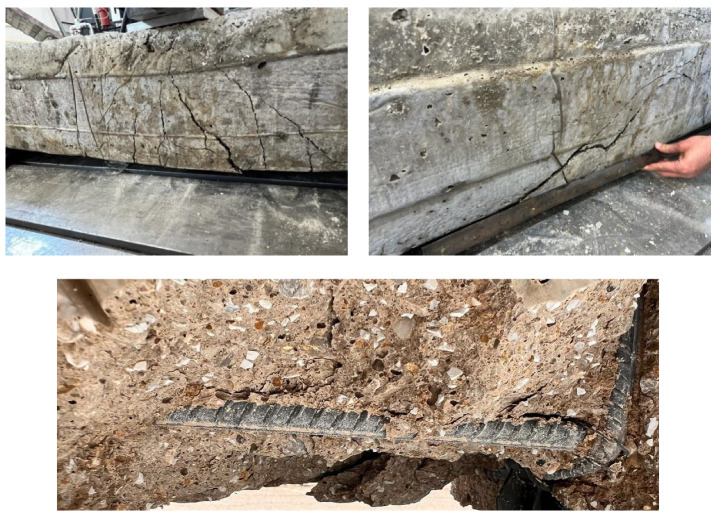
Cracking progress of the R-C-B including 15% plastic waste.

In this part of the study, the proportion of PW in the R-C-B was set as 15%. As shown in Figure 7, while the PW amount in the combination was increased from 0% to 15%, a 35% reduction in the load-carrying capability of the R-C-B was observed and the deformation proportion increased by 36%. Figure 8 shows that significant rupture occurred in the R-C-Bs, and significant slope cracking. The places of the ruptures were generally where vertical cracks could occur in the R-C-B. The load-carrying capability of this R-C-B was recognized as 14.80 kN. The resultant bending amount was 45 mm. Figure 8 presents the crack formations.

### 4.4. Case 4: Cracking and Deformation Performance of R-C-B Including 30% Plastic Waste

**Figure 9:**
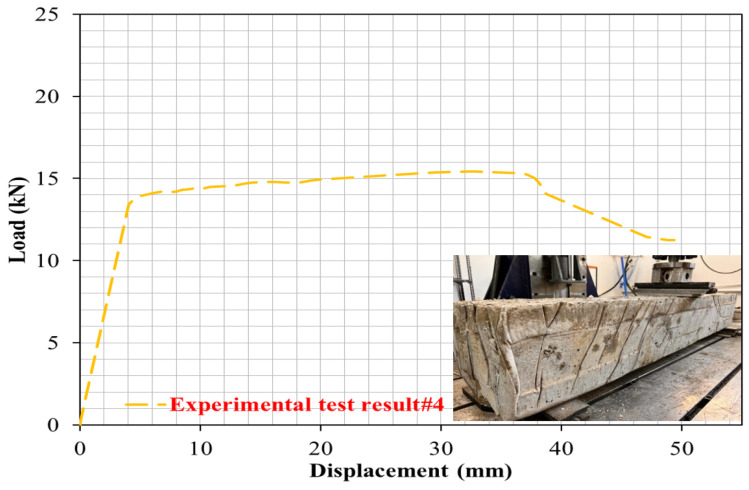
Load deviation performance of the R-C-B including 30% plastic waste.

**Figure 10:**
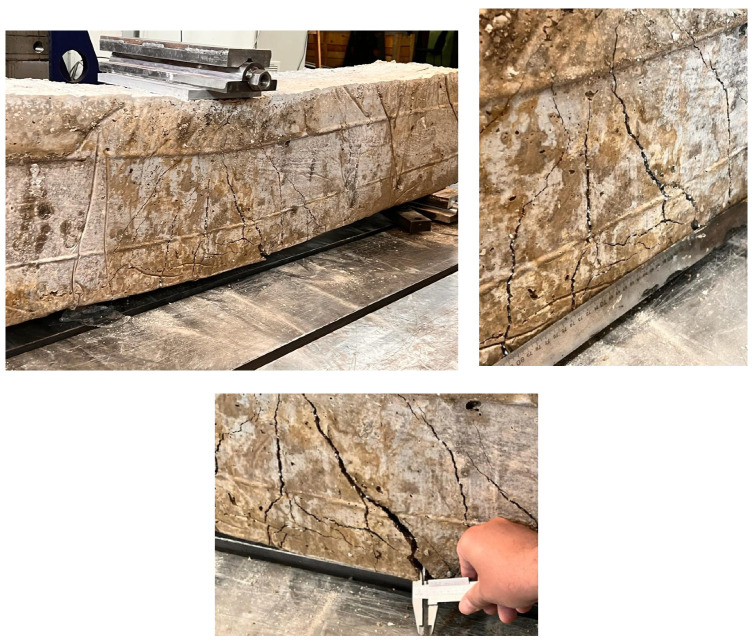
Cracking progress of the R-C-B including 30% plastic waste.

To examine the effect of PW ratio on the cracking and bending performance of R-C-Bs, the PW ratio in the R-C-B was changed to 30%. In Figure 9, with this ratio, it was observed that the load-carrying ability of the R-C-B decreased by 34%, while the deformation rate increased by 54%. In Figure 10, important cracks can be seen in the R-C-Bs, including remarkable deformation cracks. These fracture zones were observed mostly where vertical cracks could occur. It is understood from the test data that the load-carrying capability of this R-C-B was 15.1 kN. The corresponding deformation amount was found to be 51 mm. In Figure 10, the cracking patterns are shown.

### 4.5. Case 5: Cracking and Deformation Performance of R-C-B Including 45% Plastic Waste

**Figure 11:**
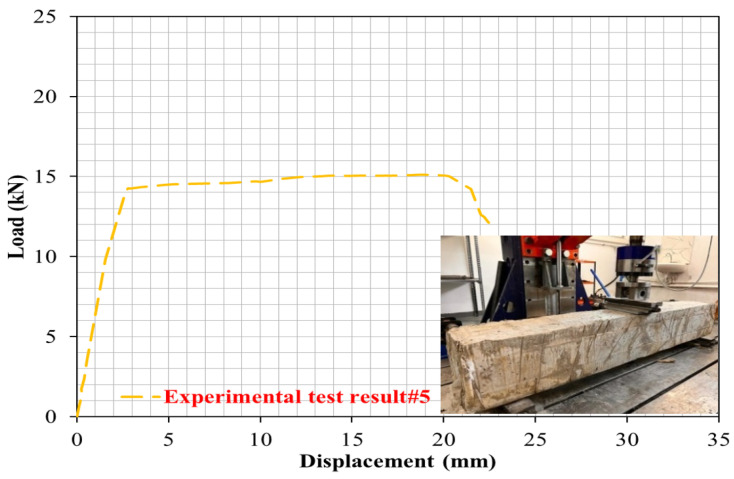
Load deviation performance of the R-C-B including 45% plastic waste.

**Figure 12:**
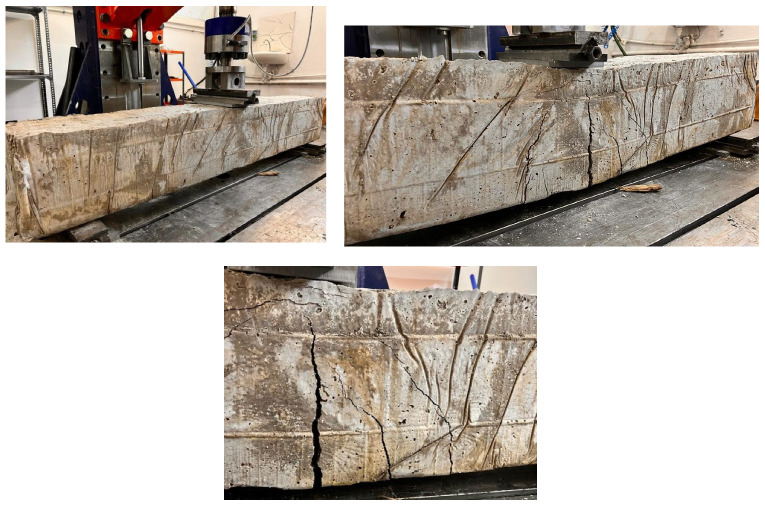
Cracking progress of the R-C-B including 45% plastic waste.

Finally, the PW ratio in the R-C-B was changed to 45%. As can be seen in Figure 11, for this mixture amount, the reduction in the load-carrying ability of the R-C-B was 33%. In addition, it was observed that the deformation rate was reduced by 33%. Figure 12 shows that a significant crack was discovered in the R-C-B. It was found that the load-carrying capability of this R-C-B was 15.3 kN. The corresponding deformation amount was found to be 22 mm. Figure 12 shows the cracking patterns.

The relationships between the load and deflection of all the R-C-Bs are shown in Figure 4, Figure 5, Figure 7, Figure 9 and Figure 11. It was observed that the results obtained were similar to the results found in the literature. It was observed that the deflection value at the same load level decreased with the presence of PW trying to close the cracks. It is worth stating that the decrease in the R-C-Bs’ deformation at first fracture with the presence of the PW was small, because the resistance of PW to tensile stresses started at the moment of the development of the fracture, while when using steel fibers, the resistance to tensile stresses starts before the presence of the first fracture, which enhances the load of the first fracture and considerably reduces the deformation of the R-C-B. Commonly, all kinds of fibers are able to bond the fractures and reorganize the stresses in the R-C-B body to move more load with smaller deformation at the same load level [22].

## 5. Three-Dimensional Finite Element Model (F-E-M) of R-C-Bs

**Figure 13:**
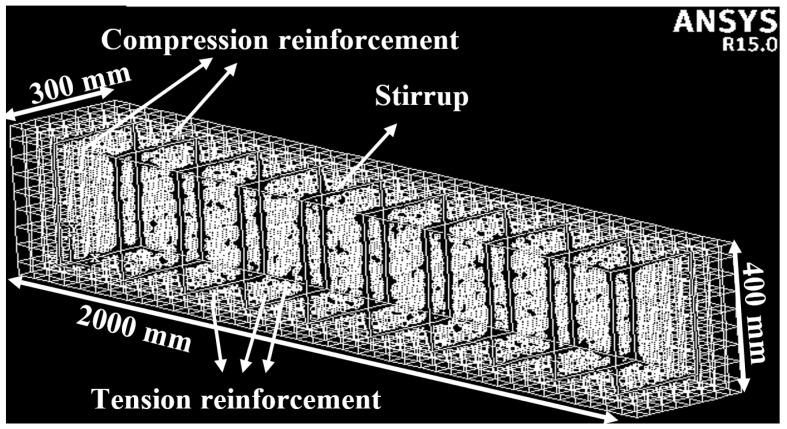
Observation of the elements in the 3D F-E-Ms of the R-C-Bs [25,31].

A 3D F-E-M is a clever approach to demonstrating cracking and deformation in R-C-Bs. Thus, 3D finite element analysis and research tests were finalized, and 3D models of R-C-Bs were made according to planned dimensions of the R-C-Bs. When constructing the 3D models of the R-C-Bs, the original R-C-B examples designed at the test site were considered. The 3D F-E-Ms of the R-C-Bs were presented via ANSYS [32] software. For five altered 3D F-E-Ms, all elements were displayed considering the original sizes of the R-C-Bs [25,31]. A total of two compression and three tension reinforcements were used at the top and bottom of the R-C-Bs [25,31]. Reinforcements with a width of 12 mm were chosen above and at the bottom of the R-C-B. Furthermore, 8 mm width reinforcements were used in stirrups. In the numerical analysis, the SOLID65 element type for concrete and LINK80 element forms and material models were used in the R-C-Bs [25,31]. Furthermore, a nonlinear multilinear von Mises plasticity material model was chosen for concrete and a nonlinear bilinear von Mises plasticity material model was elected for the top and bottom of the R-C-Bs [25,31]. The values obtained from the compressive strength were defined in the software for the creation of the 3D model. While meshing the R-C-Bs, special mesh options were chosen, and the lines were meshed first. Then, the stirrups and compression and tension reinforcements were meshed in detail. Finally, the entire mesh operation of the 3D volume was complete. The suitable mesh size for this 3D model was set as 50 mm. The 3D F-E-M for the R-C-B including all the elements is shown in Figure 13.

## 6. Three-Dimensional F-E-M Results

**Figure 14:**
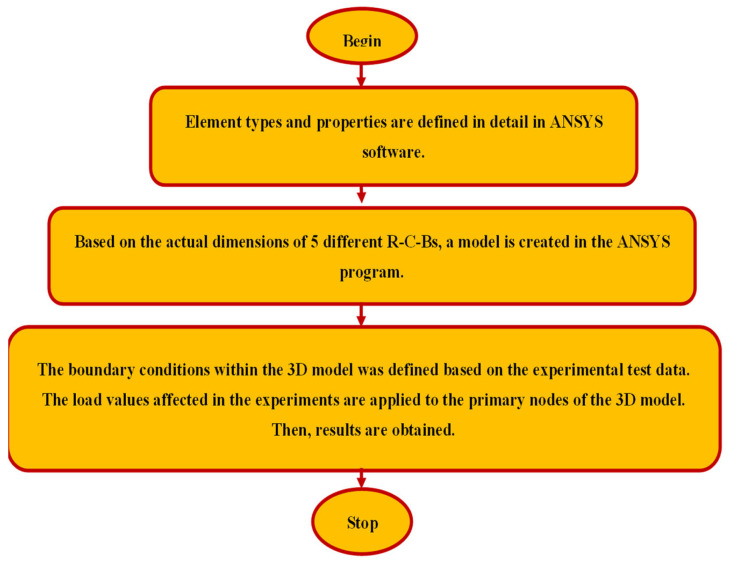
Algorithm for ANSYS studies.

**Figure 15:**
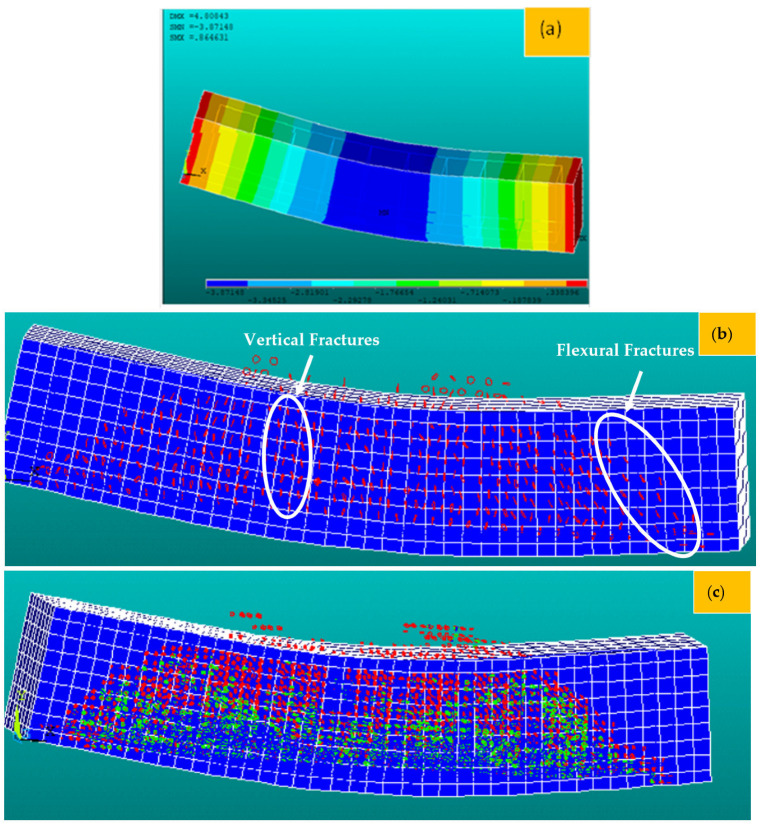
Three-dimensional F-E-M results for the reference R-C-B, containing a contour plot: (**a**) deformation; (**b**) first cracking in the R-C-B; (**c**) all cracking in the R-C-B.

In this section, the 3D F-E-M results of several R-C-Bs with PW are presented in terms of cracking and deformation performance. For this purpose, 3D examinations were conducted for R-C-Bs with five different PW proportions. The algorithm used for the numerical studies is defined in Figure 14. The 3D F-E-M results for the R-C-Bs including 0% PW are shown in Figure 15. The mid-point of the R-C-B had the maximum deviation as expected, while the edge points of the reference R-C-B exhibited the minimum deviation. The R-C-B deviation amount obtained was 32.6 mm. This result is a clear indication of how a perpendicular load affects the movement of R-C-Bs. The maximum deviation of the reference R-C-B in the experimental tests and the maximum deviation of the R-C-Bs in the 3D F-E-Ms were 33 mm and 32.6 mm, respectively. At the end of these analyses, it was found that the R-C-Bs’ maximum deviation results were similar. Cracking in the R-C-Bs was influenced by perpendicular pressure, as shown in Figure 15. This cracking was similar to the investigation results. The R-C-Bs had various large perpendicular and deformation cracks.

The 3D F-E-M results for the R-C-Bs including 5% PW were obtained from the analyses. The mid-point of the R-C-B had the maximum deviation as expected, while the edge points of the R-C-Bs including 5% PW demonstrated the minimum deviation. The R-C-B deviation amount was found to be 42.6 mm. This result is a clear indication of how the perpendicular load affects the movement of R-C-Bs. The maximum deviation of the R-C-Bs including 5% PW in the experimental tests and the maximum deviation of the R-C-B in the 3D F-E-M were found to be 44 mm and 42.6 mm, respectively. The percentage change between the test result and the 3D F-E-M result was 3%. At the end of these analyses, it was detected that the R-C-Bs’ maximum deviation results were almost the same.

Another 3D F-E-M was performed for the R-C-Bs including 15% plastic waste. As found in the earlier F-E-Ms, in this model, the mid-point of the R-C-B had the maximum deviation as estimated, while the edge points of the R-C-Bs containing 15% PW had the minimum deviation. The R-C-B deviation amount obtained was 43.6 mm. This result is a strong indication of how the perpendicular load affects the movement of R-C-Bs. The maximum deviation of the R-C-Bs containing 15% PW in the experimental tests and the maximum deviation of the R-C-B in the 3D F-E-M were found to be 45 mm and 43.6 mm, respectively. The proportion difference between the test result and the 3D F-E-M result was 2.9%. At the end of these studies, it was found that the R-C-Bs’ maximum deviation results were almost the same.

**Figure 16:**
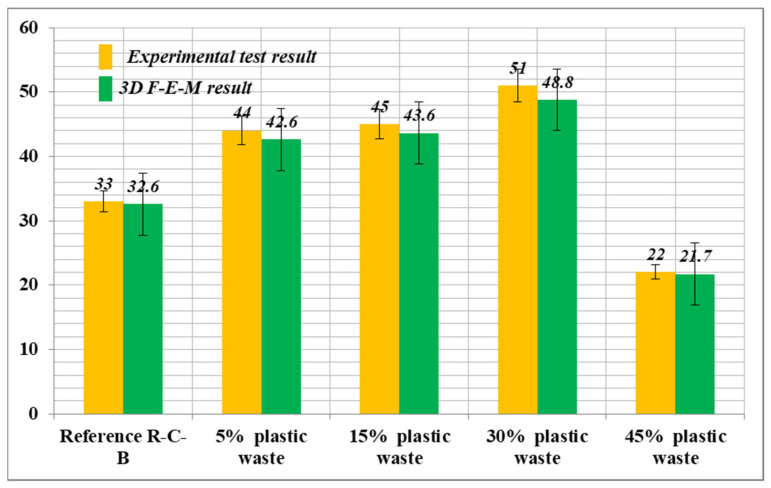
Deviation data under perpendicular force obtained through experiments and computer simulations.

**Figure 17:**
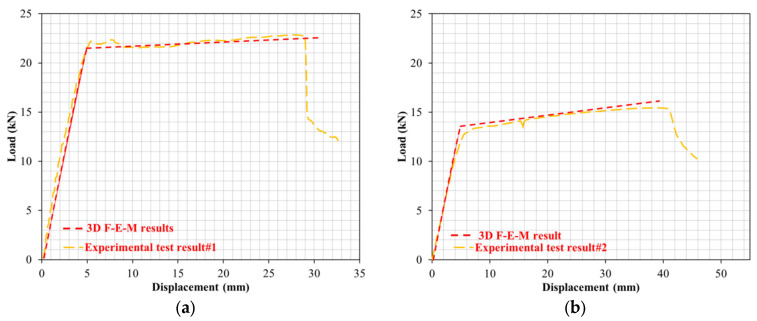

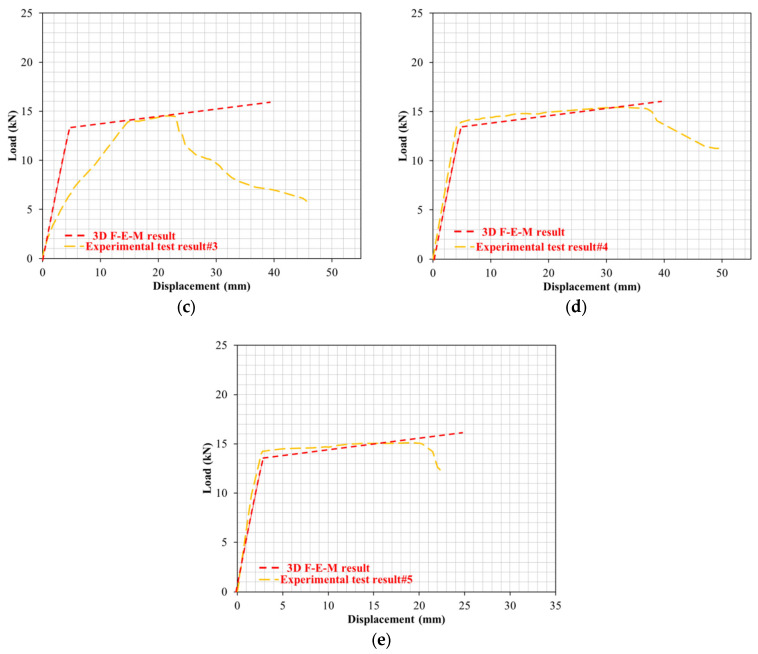
Relative analyses of 3D F-E-Ms and investigational deflection for (**a**) test#1; (**b**) test#2; (**c**) test#3; (**d**) test#4; (**e**) test#5.

**Figure 18:**
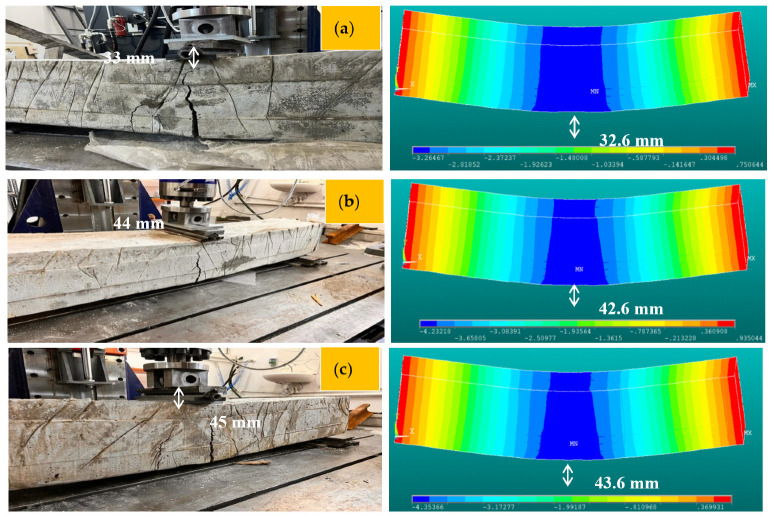

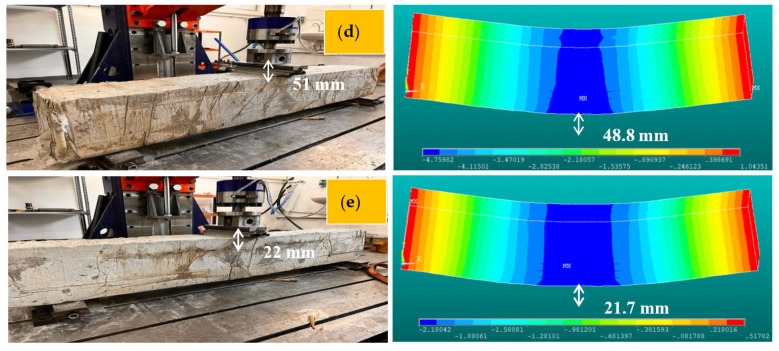
Comparison of the experimental tests and 3D F-E-Ms for (**a**) test#1; (**b**) test#2; (**c**) test#3; (**d**) test#4; (**e**) test#5.

Other 3D F-E-M analyses for the R-C-Bs including 30% and 45% PW were also conducted. As detected in these F-E-Ms, the mid-point of the R-C-Bs had the maximum deviation as estimated, while the edge points of the R-C-Bs including 30% and 45% PW confirmed the minimum deviation. The R-C-B deviation amounts were 48.8 mm and 21.7 mm for the 30% and 45% plastic waste, respectively. The maximum deviation of the R-C-Bs containing 30% and 45% PW in the experimental tests and the maximum deviation of the R-C-Bs in the 3D F-E-Ms were 51 and 48.8 mm, respectively, for the 30% PW and 22, and 21.7 mm for the 45% PW. The proportion variations among the test results and the 3D F-E-M results were 4% and 1%, respectively. At the end of these investigations, it was found that the R-C-Bs’ maximum deviation consequences were almost the same. Figure 16 demonstrates both the experimental test results and the 3D F-E-Ms for the maximum displacement in the R-C-Bs. The results of the relative analyses of the 3D F-E-Ms and investigational deflection are presented in Figure 17 and Figure 18. It was observed that the F-E-Ms and the experimental results presented small ruptures with very similar formations. In addition, it was found that the load deformation values were quite similar.

## 7. Example Study

**Figure 19:**
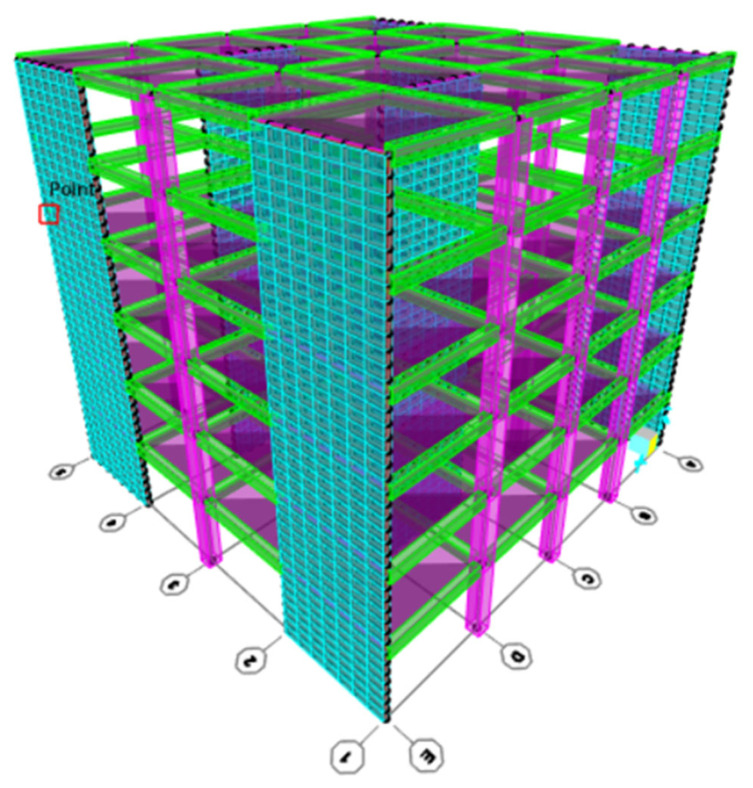
Three-dimensional model of concrete construction.

This section will demonstrate how to use SAP2000 [33] software to produce R-C-B constructions and define how numerous PW proportions influence the weight of an RC construction. This investigation also studied the effects of R-C-Bs containing 0% and 30% plastic waste. The structure was a five-story building in Turkey. The structure’s original sizes were entered into the 3D F-E-M. The resources for all columns and R-C-Bs were constructed via investigational testing on five dissimilar concrete cube series with dissimilar appearances. Figure 19 shows the 3D version of this construction.

After using SAP2000 [33] software to build the concrete construction, the two material features that were the most critical were elected to be used in the software, and the building collapsed under its own heaviness. Important weight disproportions were found in several circumstances. The concrete strength data obtained as a result of the experiment were analyzed under the total building loads and the dead loads in the building modeled on the SAP2000 program, as shown in Table 5 and Table 6. Table 5 and Table 6 are based on the most difficult section elements in the structure. As a result of the analyses, according to the modal analysis results of the SAP2000 [33] package program, the moment forces in the P0 sample were similar to the maximum forces in the P30 sample. After testing the P30 sample, it was found that the moment and shear forces of the other samples decreased; however, there was a slight decrease in the weight of the structure.

## 8. Conclusions

In this investigation, experimental tests were performed on the deformation–load performance and cracking performance of several R-C-Bs produced with plastic waste. After R-C-Bs were created in the lab, these special R-C-Bs were inspected for cracking via a special device. A perpendicular load was applied to R-C-Bs that had various PW proportions, and deformations in the R-C-Bs were calculated via an L-V-D-T. The rupture and deformation performances of the R-C-Bs were calculated in detail according to the experimental investigations. The significant results are detailed below:

- As stated by the slump test results, it was found that as the PW proportions in the concrete combination increased with a reduction in the compressive strength value of concrete after the 15% proportion.
- Based on the investigational test results, the maximum load-carrying value in the R-C-Bs reduced as the percentage of PW in the concrete combination increased. On the other hand, it was found that the maximum deformation performance of the R-C-Bs increased as the percentage of PW in the concrete combination increased.
- The percentage of PW in the concrete significantly affected the cracking performance of the R-C-Bs. Noteworthy perpendicular and deformation cracking were both seen in the R-C-Bs depending on the percentage of plastic waste.
- The F-E-M and experimental results show small ruptures with very similar formations. This suggests that F-E-M might be an excellent alternative to destructive lab experiments that can create alterations in conclusions. F-E-M simulation could be successfully used to estimate the real performance of R-C-Bs.
- The weight of a concrete construction was considered in terms of its R-C-Bs and columns. Numerous percentages of PW were chosen within the R-C-Bs and columns, and the influence of these on the construction’s forces was thoroughly defined. The forces on the construction decreased as the percentages of PW increased. The total shear forces on the constructions containing 0% and 30% PW were 853.6 and 801.07 kN, respectively.
- Considering the analyses and modeling results and the environmental effects of plastic materials, PW granules can be considered for use in concrete. In this way, environmental sustainability can be improved by preventing PW pollution. As a result of our analysis, we can state that PW granules partially contribute to concrete strength.

## Figures and Tables

**Table 1:** CEs for the plastic waste proportions.

Specimen Number	Statement
1	Reference concrete
2	5% plastic waste
3	15% plastic waste
4	30% plastic waste
5	45% plastic waste

**Table 2:** Components of the CEs and weight per unit of components (1 m^3^).

Material	Reference	5% Plastic Waste	15% Plastic Waste	30% Plastic Waste	45% Plastic Waste
Cement (kg)	415	415	415	415	415
Sand (kg)	850	805	720	595	470
15 mm lightweight aggregates (kg)	67	67	67	67	67
6 mm lightweight aggregates (kg)	333	333	333	333	333
Water (kg)	180	180	180	180	180
PW granules (kg)	0	15	45	90	135
Superplasticizer (mL)	2050	2050	2100	2300	2700
Estimated density (kg)	1845	1815	1760	1680	1600

**Table 3:** Cement composition [25,27].

Components	Weight Per Unit of Volume (%)
Portland Cement Clinker	45–64
Limestone	0–5
Gypsum	3–6
Calcium Oxide	0–5
Magnesium Oxide	0–5
Natural Pozzolan	36–55

**Table 4:** CS test results for concrete series.

Specimen	Reference (MPa)	5% (MPa)	15% (MPa)	30% (MPa)	45% (MPa)
Specimen A	40.3	41.5	42.4	33.4	29.7
Specimen B	39.6	40.8	41.6	31.6	28.5
Specimen C	40.1	43.01	39.44	28.9	30.9
Average	40	41.77	41.48	31.29	29.73

**Table 5:** SAP2000 [33] results for R-C-Bs including 0% plastic waste.

R-C-Bs Including 0%			
Modal Analysis Result			
Section	Max. Shear Force (kN)	Section	Max. Moment Force (kN.M)
F279	853.6	F248	2031.65
**Dead Load**			
**Section**	**Max. Shear Force (kN)**	**Section**	**Max. Moment Force (kN.M)**
F252	68.35	F245	64.47

**Table 6:** SAP2000 [33] results for R-C-Bs including 30% plastic waste.

R-C-Bs Including 30%			
Modal Analysis Result			
Section	Max. Shear Force (kN)	Section	Max. Moment Force (kN.M)
F279	801.07	F248	1906.62
**Dead Load**			
**Section**	**Max. Shear Force (kN)**	**Section**	**Max. Moment Force (kN.M)**
F252	64.26	F245	61.35

## Data Availability

Not applicable.
